# Effects of training podiatrists to use imagery-based motivational interviewing when treating people with diabetes-related foot disease: a mixed-methods pilot study

**DOI:** 10.1186/s13047-021-00451-1

**Published:** 2021-02-10

**Authors:** Tracey Kaczmarek, Jaap J. Van Netten, Peter A. Lazzarini, David Kavanagh

**Affiliations:** 1 School of Clinical Sciences, Queensland University of Technology, The Prince Charles Hospital Rhode Road, Brisbane, Queensland 4032 Australia; 2 Institute of Health & Biomedical Innovation, Queensland University of Technology, Brisbane, Queensland Australia; 3 Department of Podiatry, Metro North Hospital & Health Service, Queensland Health, Brisbane, QLD Australia; 4 Amsterdam UMC, University of Amsterdam, Department of Rehabilitation, Amsterdam Movement Sciences, Meibergdreef 9, Amsterdam, The Netherlands; 5 School of Public Health and Social Work, Queensland University of Technology, Brisbane, Australia; 6 Allied Health Research Collaborative, Metro North Hospital & Health Service, Queensland Health, Brisbane, Queensland Australia; 7 Centre for Child Health Research and School of Psychology & Counselling, Queensland University of Technology, Brisbane, QLD Australia

**Keywords:** Diabetic foot, Diabetes, treatment, Motivational interviewing, Imagery, Communication, Training, Podiatry

## Abstract

**Background:**

Self-care in diabetes related foot disease (DFD) is challenging and contributes to poor outcomes. Motivational Interviewing (MI) can engage people in self-care and modifying it by integrating imagery may further improve its outcomes. No previous studies have trained podiatrists in using MI to address DFD self-care. This was the first study on training podiatrists to conduct imagery-based motivational interviewing (MI) when treating people with DFD, and to examine impacts on MI related skills, job satisfaction and subjective experiences in a mixed-methods pilot study.

**Methods:**

Eleven recruited podiatrists (median age: 35 years, 9 female and 2 male) received two 4-h training sessions, and three received subsequent mentoring. MI and imagery skills were rated using validated tools during two clinical sessions per participant at baseline, and 2- and 12-weeks post-training. Job satisfaction was assessed at baseline and 12 weeks. Semi-structured interviews at 12 weeks were analysed using the framework approach.

**Results:**

Significant improvements over time (*p* = .006–.044) with substantial effect sizes (η^2^ = .50–.67) were found in three of four global MI related communication skills and two of four MI behaviours. However, effects on these indices were not sustained to 12 weeks, and imagery was rarely used. Job satisfaction was high at baseline and unchanged at follow-up (*p* = 0.34, η^2^ = .100). In qualitative interviews, MI training and skills were valued, but significant challenges in using MI when treating people with DFD were reported.

**Conclusion:**

Training podiatrists in MI may have potential but more training, observation and mentoring appear needed to obtain sustained communication changes in practice.

## Background

An estimated 451 million people worldwide were diagnosed with Type 2 Diabetes Mellitus in 2017, a number that is expected to become 693 million by 2045 [1]. A major complication of diabetes is foot disease (DFD), which includes foot ulceration [2]. Multiple pathophysiological factors including peripheral artery disease and peripheral neuropathy lead to foot ulceration [3]. Around half of these ulcers become complicated by infection, often leading to hospitalisation, amputation and increased mortality [2]. In consequence DFD is a leading cause of the global burden of disability [4, 5]. However up to 75% of this foot ulceration may be prevented when evidence-based clinical care is optimised [6].

Optimal evidence-based care of DFD often relies upon sustained engagement to recommended self-care [7], such as regularly changing ulcer dressings, monitoring for signs of infection and wearing offloading devices [8]. Maintaining optimal self-care is extremely difficult for people with DFD to achieve [8–11]. Education can improve knowledge and self-care in the short-term, it has yet to show longer-term benefits [12]. A traditional “physician-directed” style of education and communication by clinicians, where people are told to adhere to recommended behaviours [13, 14], may undermine commitment to effective change of behaviour for engagement to self-care if it elicits arguments in favour of the status quo [13]. However, practitioners may help people with DFD engage with self-care and facilitate ulcer prevention and healing when they build collaborative relationships [9, 15, 16]. A paper looking at how podiatrists provide education found various methods to be used in communication with people with DFD [17]. However, the use of specific communication techniques or relationship building were not reported in their survey [17]. One such technique to establish a relationship with people with DFD is motivational interviewing (MI) [18].

MI is an evidence-based approach that enhances motivation for functional behavioural change by helping people resolve ambivalence [15, 19]. Its empathic and accepting manner provides a safe setting to consider behaviour change, while its agenda encourages exploration of the advantages and possibility of improved self-care [20]. MI has been successfully used in behaviour change in healthcare, including prevention of Human Immunosuppressive Virus, modification of substance abuse and improved outcomes in diabetes [19, 21]. However, not all studies report substantial and sustained success (e.g. [22]).

A recent modification and extension of MI involves incorporating mental imagery throughout sessions [23, 24]. Mental imagery is more closely linked to emotional reactions than is verbal discussion, and the affect is central to the experience of being motivated [25]. In Functional Imagery Training (FIT), participants are encouraged to use individually tailored mental imagery when considering the benefits of improved self-care, and when remembering past successes [23, 24]. If they become committed to changing their behaviour, they create mental images about their plans, and rehearse those at home to motivate and guide their self-care. An early form of this approach was tested in people with type 2 diabetes and showed substantial acceptability [26]. A multi-session version has shown stronger outcomes than standard MI in dieting, exercise and weight reduction [27, 28].

Effective application of imagery-based MI requires that practitioners are sufficiently trained to apply it routinely with high fidelity in their work [19]. MI skills can be acquired by nurses or general practitioners who care for people with diabetes, albeit to varying success [22, 29–33], depending in part on the length of MI training and availability of supervision [34]. However, training podiatrists in using MI to address DFD self-care has not been investigated yet. Podiatrists are in a prime position to engage people with DFD in carrying out self-care, and training podiatrists in imagery-based MI could be a valuable addition to their clinical skills [35]. An observational study of podiatrists working with people with DFD identified that 90% perceived their role in promoting self-care as vital, but only 25% reported use of “MI type” skills [36]. Increasing this level represents an acute need.

Our aim was to train podiatrists to conduct imagery-based MI when treating people with DFD and use a mixed methods approach to obtain pilot data on its effects. We assessed the impact of the training on the skills podiatrists showed in routine sessions. Since we were aware that lack of apparent engagement in effective self-care by people with DFD was a source of significant frustration for podiatrists, we examined whether the training also had effects on job satisfaction. Quantitative study of the podiatrists’ experience of training and attempted application of the skills was obtained to provide a deeper understanding of the effects of training and of any changes that may be needed in future research.

## Methods

This mixed-methods pilot study involved podiatrists who primarily treated people with DFD. The design was chosen to obtain outcomes on a variety of measures, to best assess the outcomes of training and inform future training implementation and investigation. Its quantitative component comprised a single-group pre/post design, testing the effects of a training program on MI and FIT-related skills of participants at baseline, 2- and 12-weeks post-training, and self-reported job satisfaction at baseline and 12 weeks. The qualitative component comprised a semi-structured interview at 12-weeks post-training, to obtain information regarding podiatrists’ experiences of the MI training and of their attempts to apply it in practice. The study received human ethical approval from the health service and university (HREC/2018/QPCH/45318). The Template for Intervention Description and Replication (TIDieR) checklist was used for the reporting the methods (Appendix A).

### Participants

Participants for this study were consenting registered podiatrists who worked within a single government funded podiatry team from Metro North Hospital and Health Services in the city of Brisbane, the state capital of Queensland, Australia. podiatrists in this team worked in community settings and primarily treated people with DFD (i.e. with a diagnosis of diabetes and a history of foot ulceration) The first author was a senior member of the team, which facilitated both team engagement and management approval for the training and practice changes”. Exclusion criteria included prior training in MI. All podiatrists in this team were informed about the study by recruitment emails and posters displayed in clinical rooms.

Participating podiatrists recruited people with DFD to assist with outcome assessment. These people also provided voluntary written consent to participate in recorded clinical sessions during which podiatrists’ skills in imagery-based MI were assessed.

### Intervention

The training was planned in collaboration with the manager of the podiatry team, who gave the project strong support, linked training sessions to team meetings and actively participated in the training sessions. The intervention training program was jointly developed and delivered by two authors: a clinical psychology researcher (DK) and a senior podiatrist in the trained team (TK). DK is a co-developer of FIT and has led multiple controlled trials using MI or FIT [27, 37, 38]. TK was trained in MI and FIT before study commencement and had experience applying these skills in her own clinical sessions, allowing her to incorporate detailed descriptions of her experience in training.

The program comprised two 4-h face to face training sessions in imagery-based MI, separated by 2 weeks. Training was delivered in groups of 5–6 participants a log of these training sessions describing the core outline is attached (Appendix B). The first session included group discussion of issues participants had experienced in attempting to facilitate DFD-related self-care, and of advantages for morale of defining success in terms of delivering a favourable context for change, rather than reaching behavioural or clinical targets. It outlined core concepts of MI and provided video examples of MI, followed by group discussion of potential application in podiatry sessions. A flowchart gave some suggested prompts for podiatrists to use in consultations. To maximise compatibility with existing sessions, trainers suggested incorporating elements of MI when assessing self-care, conducting physical treatment and collaboratively planning future self-care. In line with the International Working Group on the Diabetic Foot (IWGDF) recommendations and definitions, self-care included, but was not limited to, engagement in keeping dressings dry, wearing offloading devices and consistent use of suitable footwear, as these are the main self-care activities targeted by podiatrists [39].”

The second session described the use of imagery and home-practice in podiatry sessions, with demonstrations, roleplayed practice and feedback to build skills and confidence.

All participants were offered follow-up peer support opportunities from TK to observe and feedback on consultation sessions or provide peer-mentoring via email, phone or face-to-face. Such feedback and advice were positive and practical, recognising incremental improvements and helping participants solve challenges in their application of MI related skills. Emails reminding participants about key elements of the intervention were sent 4 and 10 weeks after the workshop training.

### Proposed context for use of MI by podiatrists

The aim of the training was that podiatrists would routinely deliver MI to everyone with DFD who they treated. It was envisaged that MI would be incorporated within usual treatment sessions, which last approximately 45 min for a review appointment or 60 min for a new appointment, and are delivered individually. There was no opportunity to provide extended sessions: instead, MI was intended to be given in conjunction with assessments and physical treatment. Accordingly, the training and form of MI were customised to reflect this delivery mode. All information given in these sessions were as per standard care so people with DFD were not advised to expect appointments to be any different than usual. In most cases in an oral format unless written instruction was required for individual people.

### Outcomes of interest

The outcomes of interest for the quantitative component included MI related skills, use of imagery and job satisfaction. Validated instruments were used to assess MI related skills and imagery, using audio recordings from two clinical consultation sessions by each podiatrist at baseline, and 2- and 12-weeks post-training. Follow up at 2 and 12 weeks was chosen to investigate changes immediately following training, and at longer follow-up. With the challenges of care for people with DFD, we hypothesized that training effects would not be visible at 6 or 12 months (as used in other studies [29, 30, 32, 33, 40–42]), hence long-term assessment at 12 weeks was chosen to provide rationale for the timepoints chosen in our research methods. Sessions were selected opportunistically, based on availability of the participants and researcher. No data on characteristics of the people with DFD or outcomes of sessions were collected, since the focus was on podiatrists’ conduct of sessions. In order to not require any changes to routine practice, a relative was able to be present in the coded interviews. This only occurred in three of the 66 total recordings and the relatives were asked to remain silent. While it is possible that their presence may have altered the interchange with the podiatrist, in only one case did the recording have to be terminated because the relative’s contributions interfered with the conduct of MI. Accordingly, the audio recording of that session was discontinued, and an additional session recorded and then used for coding.

MI related skills were assessed using the Motivational Interviewing Treatment Integrity Tool (MITI) 4.2.1 [43]. MITI has two components: global scores and behaviour counts. Four global characteristics (Cultivating Change Talk, Soften Sustain Talk, Partnership, Empathy) are each scored from 1 (low) to 5 (high). Three core MI adherent behaviours (Affirm, Seeking Collaboration, Emphasizing Autonomy), two non-MI adherent behaviours (Persuade, Confront), and five other relevant behaviours (Giving Information, Persuasion with Permission, Questions, Simple and Complex reflections) were counted. Higher counts in Questions and Information Giving were expected, since consultations had to include clinical assessment and instructions for foot care. Consistent with MITI instructions, only the first 20 min of each session were assessed. Assessment of clinical sessions were undertaken independently by two authors (TK and DK), who reached consensus after discussion and replay of audio segments where required.

FIT-related skills during the same session segments were assessed using the Functional Imagery Training – Quality Coding (FIT-QC) 1.0 [44] which includes introduction to imagery, instruction, delivery, use and adaption of imagery, refinement of imagery quality and focus, reflection and promotion of home practice. Each component was scored from 1 (poor) to 5 (high). FIT skills were assessed by the same authors (TK and DK).

Job satisfaction at baseline and 12-weeks post-training was assessed using the Hoppock Job Satisfaction Scale [45], which has 4 items rated 1 to 7, with higher total scores denoting higher satisfaction [45]. The rationale for measuring job satisfaction was that podiatrists had expressed frustrations with lack of engagement from people with DFD. Hence, we wanted to explore if the use of skills to improve this engagement would result in improved job satisfaction.

Individual face-to-face semi-structured interviews at 12 weeks post-training were undertaken by the primary author (TK), audio recorded and transcribed verbatim. The interview was designed to gain insight into the podiatry participant’s experiences in training and their application of MI skills into clinical practice. Each primary question in Table 1 was augmented by non-specific questions to obtain additional responses.

**Table 1:** Questions used in the Semi-Structured Interviews

Interviewer Question	
Before the training, what aspects of working with your patients did you find most challenging?	
Have you noticed any differences in your patient’s responses since doing the training?	
What was your experience of the training like?	
What aspects of training seemed most useful for your practice?	
What aspects of the training seemed least useful for your practice?	
What aspects of the training were easiest to apply into clinical practice?	
What particular aspects were more difficult to apply?	
What challenges did you face in applying what you learned in the training?	
What could we do to help you keep using your skills from the workshop routinely with your patients?	

### Analysis

Quantitative data were analysed using repeated measures general linear models on IBM SPSS v26, with *F* values using Pillai’s Trace. The primary focus was the overall effect for time, but nonorthogonal contrasts examining changes from baseline to 2- and 12-weeks post-training were also examined. Because this was a small pilot, η^2^ (the proportion of total variance accounted for by an effect) was the primary focus rather than statistical significance. However, we have also reported probability values for descriptive purposes, in the interests of full disclosure. The reported effect sizes inform potential future studies on sample sizes that may be required to detect effects.

Qualitative interviews were analysed using the framework approach [46] which was chosen as the method best fitted with our aims [46]. After transcription of interviews, participants’ responses were analysed using: 1) familiarisation (re-reading transcripts and field notes), 2) identification of the thematic framework (identifying key themes from data), 3) indexing (arranging transcripts by themes), 4) charting (creating a matrix with responses collated), and 5) mapping and interpretation (interpreting the data). Initial themes were identified by one author (TK). A second assessor (JvN) reviewed all transcripts and identified the themes independently. The assessors then discussed the themes until consensus was reached.

## Results

Of 14 eligible podiatrists who were approached, 11 consented to participate. Of the remaining three, one was on vacation, one did not express interest and one expressed interest but did not consent. Participants were aged between 29 and 43 years (median 35), nine (82%) were female, and years of DFD-treatment experience ranged from 2 to 17 years (median 11). Two training sessions were delivered to all participants. Five participants requested peer support following training and three of these received feedback about a clinical consultation session. The amount of peer support ranged from 0 to 4 h.

Three of the four global score ratings (Change Talk, Partnership and Empathy) and two of the four core behaviour counts (Affirm and Persuade) showed statistically significant improvements and substantial effect sizes (η^2^ = .50–.67) across the three time points (Table 2). Contrasts comparing scores with those at baseline showed significant improvements on these six indices 2 weeks but not 12 weeks post-training.

**Table 2:** MI related skills at baseline, 2- and 12-weeks post-training

				Overall Time Effect			Time Contrasts					
				from Pillai’s Trace			Baseline – 2 weeks			Baseline - 12 weeks		
Variable	Baseline	2 weeks post-training	12 weeks post-training	*F* [2, 9]	*p*	η^2^	*F* [1, 10]	*p*	η^2^	*F* [1, 10]	*p*	η^2^
	M (SD)	M (SD)	M (SD)									
Global scores												
Change Talk	1.77 (0.79)	3.08 (1.28)	2.08 (1.08)	5.60	.026	.554	10.92	.008	.522	0.79	.395	.073
Soften Sustain	1.09 (1.46)	1.36 (1.64)	1.00 (1.32)	0.14	.874	.030	0.20	.665	.019	0.02	.883	.002
Partnership	2.21 (0.74)	3.08 (0.96)	2.36 (0.98)	9.30	.006	.674	6.78	.026	.404	0.19	.671	.019
Empathy	2.34 (1.18)	3.09 (1.04)	2.27 (1.03)	6.91	.015	.605	3.31	.099	.249	0.03	.859	.003
Focal MI behaviour counts												
Affirm	0.68 (0.93)	1.23 (1.08)	0.64 (0.81)	6.12	.021	.576	2.68	.133	.211	0.02	.905	.001
Seeking Collaboration	1.65 (1.68)	2.91 (2.33)	2.56 (2.12)	1.66	.243	.270	3.69	.084	.270	1.74	.216	.148
Emphasising Autonomy	0.05 (0.15)	0.36 (0.55)	0.36 (0.50)	2.00	.192	.307	3.06	.111	.234	4.22	.067	.297
Persuade^a^	1.36 (1.12)	0.41 (0.66)	2.36 (2.60)	4.52	.044	.501	4.51	.060	.311	1.43	.260	.125
Other behaviour counts												
Giving Information^a^	19.30 (8.55)	13.41 (5.13)	11.05 (6.04)	3.36	.081	.428	3.50	.091	.259	7.12	.024	.416
Persuade with Permission	0.00 (0.00)	0.14 (0.32)	0.14 (0.24)	2.43	.143	.351	1.96	.192	.164	3.75	.082	.273
Question	19.55 (7.92)	18.89 (5.25)	18.64 (5.80)	0.58	.944	.013	0.07	.798	.007	0.13	.731	.012
Simple Reflection	1.36 (1.23)	2.38 (1.70)	0.76 (0.50)	4.14	.053	.479	3.13	.107	.239	2.05	.183	.170
Complex Reflection	0.18 (0.34)	1.14 (1.19)	1.05 (1.65)	3.99	.057	.470	6.58	.028	.397	2.87	.121	.223

Note: ^a^Lower Persuade or Giving Information scores indicate better MI adherence. The non-adherent behaviour ‘Confront’ was not seen in any recordings and therefore omitted from the table. *M* Mean. *SD* Standard Deviation

None of the five other behaviour counts showed statistically significant improvements, but Simple Reflections, Complex Reflections and Giving Information each showed moderate effect sizes (η^2^ = .43–.48). Only Giving Information showed a statistically significant improvement from baseline to 12 weeks. As anticipated, both Giving Information and Questions were frequent throughout the study.

No substantial use of FIT Skills was evident in recordings, with only one podiatrist attempting to use motivational imagery after the training (by asking a patient to imagine what it would be like to have no foot ulcer). Accordingly, detailed results on the FIT-QC are not reported.

Ten podiatrists completed the Hoppock scale. Job satisfaction was high at both baseline (Mean = 19.4, SD = 3.2) and 12 weeks (Mean = 20.4, SD = 3.9), with no significant change over time (*F* [1, 9]=1.00; *p* = 0.34, η^2^ = .100).

Semi-structured interviews were conducted with all 11 participants and lasted a median 5.5 min (Range = 5.1–10.4). Three main themes with 10 subthemes were identified from the interviews (Table 3).

**Table 3:** Main themes identified in semi-structured interviews

Main Theme	Subtheme 1	Subtheme 2	Subtheme 3	Subtheme 4
**1. Clinical Issues**	1.1 Challenging situations	1.2 Communication challenges		
**2. Training Content**	2.1 Overall training experience	2.2 Role-play	2.3 Imagery	2.4 Ongoing training and support
**3. Training Outcomes**	3.1 New communication skills	3.2 Increased patient engagement	3.3 Long-term application of MI skills	3.4 Appropriateness of MI

### Main theme 1. Clinical issues

#### Subtheme 1.1 challenging situations

Before training, all podiatrists identified struggles with fostering self-care by people with DFD. These struggles included situations where podiatrists tried to achieve commitment to self-care. *(In this subtheme, participants were specifically referring to problems before the training. However, as also seen in the subsequent themes, some identified problems were still present.)*

*“ … a bit like that feeling like your hitting your head against a brick wall. That you know they know what they should be doing and they’re just not doing it and it’s frustrating to be able to have the solution but them not being invested enough in their own care to do it”* (Pod B)

#### Subtheme 1.2 communication challenges

Podiatrists also reported challenges with communication, especially when the person had multiple comorbidities or limited education, and when relatives or friends joined consultations.

*“The relatives and the family, because it becomes a joint problem a joint concern … some of the carers have sort of pushed their needs onto the patient and you have … to steer the consult back to the patient sometimes.”* (Pod F).

### Main theme 2. Training content

#### Subtheme 2.1 overall training experience

Most podiatrists reported that MI training was enjoyable, beneficial, interesting and informative. They liked the small group and felt engaged.

*“I found the training days very beneficial and informative, and I think that we were very engaged. It was quite good because it was interactive.”* (Pod G).

*“It was good … .I definitely did learn better communication skills to a certain extent”* (Pod K).

Some felt:

*“There was a lot of information in a short period of time”* (Pod C)

*“Hard to relate it sometimes to a clinical setting”* (Pod D)

#### Sub theme 2.2 role-play

Role-play practice elicited mixed feelings. Some found it useful, despite some negative feelings.

*“Nice to see how different clinicians worked.”* (Pod A).

*“As much as I hate role-play, it did help.”* (Pod I).

Others said:

*“[Role-play is] tricky as we think differently than the patients.”* (Pod J).

*“[I] hated it.”* (Pod B).

#### Sub theme 2.3 imagery

Participants struggled fitting imagery into their clinical practice, predominantly because it was very different to clinical practice:

*“I found imagery most difficult to implement in a clinical environment. I thought we were reasonably well trained in it, but when I tried to execute it, I found it difficult to approach that kind of a thing with the patients. And when I did, they weren’t particularly receptive.”* (Pod C)

#### Subtheme 2.4 ongoing training and support

More training was requested, via ongoing support in the form of peer support and via hands-on training where actual difficult patient situations can be discussed:

*“If we were able to maybe isolate some of the difficult patients and have a group discussion about how you would apply that training to that particular patient. Then everyone can come away with a new way to deal with similar patients.”* (Pod C)

### Theme 3. Training outcomes

#### Subtheme 3.1 new communication skills

Applying new strategies when communicating with people with DFD, such as reflections and open-ended questions, was seen as useful.

*“ … having those open-ended questions other than short-ended questions, so you could really just learn to shut up and let the patient speak.”* (Pod E)

“*Strategies of getting the patient to commit themselves to something as opposed to us dictating to them what they needed to do.”* (Pod E)

Participants liked the fact that training allowed them to ask people with DFD to reflect on what they had previously done and wanted to do:

*“I think the open-ended questions and the reflections back were really useful for developing a rapport with the patients.”* (Pod D)

Other useful skills were asking for goals, allowing the patient to talk more, and reducing podiatrists talk time:

“*I have allowed the patients to speak more which has given me an insight that I did not have before*” (Pod F).

*“To be quiet as well, that’s a hard one. It’s a very practical thing to let them just speak, to fill the silence rather than us.”* (Pod B).

*“Getting the patient to tell you what their plan is and what their goals are, because at the end of the day it’s their health care.”* (Pod A).

#### Subtheme 3.2 increased patient engagement

The new communication skills appeared to result in increased patient engagement:

*“They’ve seemed to be more engaged with their self-care. And instead of telling them what to do I feel that they respond better because they are thinking what they can do better. They’re taking responsibility more.”* (Pod A)

*“Initially getting people to open up and talk about experiences with foot ulcers and how they can change things, its changing their mentality from them thinking that its actually our problem and our responsibility to them actually having to think about it as their wound and their responsibility.”* (Pod G)

#### Subtheme 3.3 long-term implementation of MI skills

Participants reported difficulty with changing their habitual behaviour in sessions. While anticipating that applying MI over the longer-term would be hard, some were optimistic about achieving this.

*“ … it was definitely still challenging. Hard not problem-solving”* (Pod D)

Participants suggested ways to support them to sustain their use of MI related skills over the longer term:

*“[We need] more training within motivational interviewing to keep your skills up and keep it fresh. The more you do it, the more it will embed into your practice, but at the beginning it’s easy to go back to how you used to do.”* (Pod A)

*“We slowly slip back into our old ways because we don’t reinforce it and we’re really time poor.”* (Pod F)

#### Subtheme 3.4 appropriateness of MI

MI was not seen as an appropriate approach for every patient:

*“Some of them still do the same thing regardless.”* (Pod J).

*“I guess I use it where I see the need.”* (Pod I).

## Discussion

This was the first study to investigate the impact of delivering MI training to podiatrists who treat people with DFD. The training resulted in some moderate short-term improvements in MI related skills after 2 weeks, but no improvements remained at the 12-week follow-up, and only one podiatrist attempted to use imagery in the assessed sessions. Improvements in job satisfaction were not observed, although satisfaction was already high at baseline and the training did not encompass broader issues related to the work setting.

The qualitative responses were largely consistent with other studies. Participants saw the interactive nature of workshops as beneficial, although some expressed discomfort about role-play [47]. Participants had positive experiences with engaging and empowering patients after training, as also found in another study [48], but expressed a need for ongoing supervision and support and noted the risk of reverting to old habits of traditional advice giving [40].

The short-term improvements in MI related skills of Empathy [30, 31, 49, 50], Change Talk [22, 30, 51] and Partnership [29] were consistent with changes in previous studies on MI training. Reductions in Persuasion are less often reported explicitly [32]; in the current study, this behaviour appeared to return to baseline levels at 12 weeks. The skill Soften Sustain did not change during the study, but statements requiring this type of response by podiatrists were surprisingly rare in the assessed audios. Rises in Simple and Complex Reflections were small in number, and effects for time fell short of statistical significance, suggesting that they require further attention in training. While both Questions and Giving Information were very frequent, these were required elements in the podiatry sessions, and it is not clear what would be an ideal frequency. The 43% reduction in Giving Information was encouraging, if it reflected a reduction in the repetition of information within and across consultations, since such repetition runs the risk of undermining rapport and collaborative self-care.

Participants’ positive experiences and short-term MI related skill improvements provide a solid basis for further changes in practice. However, the lack of maintained changes in core MI skills and reported difficulties with their routine use suggest that the training and other support that was provided in this study may have been insufficient to maintain practice changes. Among other improvements to the intervention, greater attention to the engagement of the podiatrists and additional skills training including follow-up peer support session observation and feedback may be needed. Sustained engagement and use of the skills by podiatrists may also require ongoing feedback on improvements in self-care and DFD outcomes additional training, including follow-up peer support session observation and feedback. The qualitative responses suggested this would be highly valued. With 8 h of training provided in the current study, half of the median 16 h found in a systematic review [34], more training may indeed be needed for lasting positive effects on practice. In particular, further group discussion of difficulties experienced in applying MI is recommended. Difficulties in maintaining the use of new skills has often been observed in other studies on practitioner training [22], including training on MI. A reliance on workshops alone typically gives limited skill improvement [52] and little sustained benefit [40, 41, 53]. Monitoring accompanied by encouraging and corrective feedback appears critical [40, 41, 48, 53, 54], at least until use of the skills becomes habitual. The positive short-term training outcomes and positive evaluation in this pilot study suggest that podiatrists are receptive of more intensive training, can be seen as an encouragement for further research and implementation.

A strength of this study was our incorporation of several principles of implementation science to facilitate training uptake [55, 56]. The project had strong management support, and one of the authors (TK) was a senior member of the team and modelled early adoption of the approach in her own work. Further, the training highlighted incentives for use of MI, including the potential for MI to reduce podiatrists’ guilt or frustration by defining success in terms of their conduct of sessions rather than clinical or behavioural outcomes. Finally, we minimised change from existing practice by integrating MI with assessment, physical treatment and planning within the existing session length.

Despite these facilitators, podiatrists had difficulties integrating MI into clinical sessions if these required substantial clinical assessment and physical treatment. This integration probably needed more training than we were able to deliver, and for some attempting to use a new skill while conducting another task may have been too difficult. Podiatrists were also discouraged when people with DFD did not respond positively, and additional encouragement to persist in the face of this is required. The complexity of people’s co-occurring problems also presented significant challenges for use of MI, as did addressing the needs of carers.

A limitation of this novel pilot study was the small sample size. However, we recruited sufficient participants to provide estimates of potential effect sizes for the planning of future randomised controlled trials, as well as important findings on training content and implementation. While the opportunistic selection of podiatrists by recruitment from one hospital and health service team raised issues of representativeness, it allowed the development of group processes that fostered the team’s acceptance of the innovation. Similarly, the involvement of a senior member of the team in both the training and assessments may have introduced bias, but as already noted, it also assisted with management approval and gave her authority as a trainer and mentor. In addition, the use of validated tools and independent ratings by two assessors reduced the risk of bias in assessments. However, establishment of the utility of MI training for podiatrists will require its demonstration in multiple settings and in controlled studies that have assessors who are blinded to condition. Another limitation and potential source of bias concerns selection and small number of recordings for each podiatrist (two each). While podiatrists indicated that they did not take patient characteristics or other factors into account when negotiating their availability for session observation, their involvement in session selection and the limitation of interviews to two per measurement occasion is a limitation. As collecting patient information was outside the scope of the current project, we were unable to formally assess if patients who were seen during these recordings were representative of daily practice.

A further potential limitation was involvement of the trainers in the conduct and rating of assessments, which may result in assessment reactivity and rating biases. We minimized this by using validated tools, and having two assessors working independently. Qualitative interviews were short, but derived rich data on participants’ experiences, although only limited answers were provided concerning experiences with FIT, and we did not want to ask leading questions to maintain the open approach of the interviews. While these interviews were held by a fellow team member, podiatrists indicated they were not holding back their views, and the use of a second assessor helped to ensure that the bias produced by an existing relationship did not influence the rating of sessions.

The qualitative interviews and the quantitative results suggested that inclusion of mental imagery in the initial MI training may have been premature. Participants found it difficult to introduce and use in sessions, and their discomfort in using it may have undermined their uptake of core MI skills in their practice. The observation that mental imagery was thought to be inconsistent with usual podiatry practice was an important finding of this study, and was in contrast to previous training experience by the last author, which have typically been with practitioners with more prior experience in conducting verbal therapies [23–25]. In retrospect, improvements in MI-related skills should have been consolidated before introducing mental imagery. Not only would more training on introducing and using imagery in a clinically applicable and relevant way be needed, but it may also have to be preceded by discussion about its relevance to and consistency with podiatry practice.

Training was given in conjunction with regular meetings of the podiatry team, which maximised attendance. Participants agreed to have interviews recorded, but mentoring could not be made mandatory in this study. We recommend that mentoring be more integrated with the other training, that a commitment to engage in mentoring sessions be obtained at the start of the training, and that additional positive experiences of performance feedback be given during the primary training sessions to reduce anxiety and elicit positive expectations of the later sessions. Reasons provided by participants for not accessing peer support was due to clinical case load not allowing for time to arrange mentoring sessions. This supports the idea of having pre-arranged feedback time as part of standard training.

This study did not aim to measure outcomes of improved self-care for people with DFD, as our aim was to first ensure that podiatrists were trained proficiently in MI. Investigating such clinical outcomes in this population, and investigating if MI is more applicable in relation to certain self-care activities (e.g. wearing offloading devices) compared to other activities (e.g. dressing changes), remains a topic for future studies.

## Conclusion

We provided 8 h of valued training in MI to podiatrists who treat people with DFD. This resulted in some uptake of MI related skills, although maintenance of these changes was short lived. Findings of this study provide a solid basis for refinement of the training and support for implementation and future research of its impact on both podiatry practice and outcomes for people with DFD.

## Appendix B

### Training Log

**Training session 1:**

Monday 4th of March 2019, (6 participating podiatrists) and Friday 8th of March 2019, (5 participating podiatrists).

- Welcome and introduction from Professor Kavanagh (DK), including his background experience.
- Invited podiatrists to introduce themselves to the group and say what they hope to gain from the training session today.
- Presentation of some slides around motivational interviewing and techniques.
- Flowchart read through with examples of use from DK.
- Video examples of poor communication followed by communication using MI in practice.
- Role play examples of MI and FIT by DK and Tracey Kaczmarek (TK), followed by group discussion.
- Practice role play in pairs for podiatrists; with observation and feedback from DK and TK.
- Discussion around scenarios and current ambivalence of people with DFD under podiatrist care
- Discuss role play – scenarios worked through, tools used and difficulties
- Role play between DK and TK re ambivalent person with DFD.
- Discussion regarding role play just demonstrated
- Further role play practice by podiatrists
- Summary of the session

After the session a thank you and reminder email was sent to podiatrists which thanked them and acknowledged their input in this new venture. Reminded them to work through the flowchart in the next week in clinical sessions as suitable and feedback at the next session.

Training session 2:

Monday 18th March 2019 (6 podiatrists attended) and Friday 22nd March 2019 (5 podiatrists attended).

- Welcome and request for feedback of experiences using flowchart over the last week.
- Video examples of MI in use.
- Group discussion after the video regarding the skills that were used – highlighted the positive exchange between clinician and person with DFD and the environment and support to facilitate change as needed and wanted by person with DFD.
- Read through flowchart as a group working through examples
- Role play practice by podiatrists with observation and feedback from DK and TK
- DK talked about the need for short term goals to be found for people with DFD. Short term goals decided upon by group included;
- Sense of achievement
- Less dressing changes for person with DFD to do themselves
- Less visits to GP /Pod – do nicer things with their time and not having to rely on people to bring them
- More independence
- Less cost to the person with DFD of non-government services to come out and do dressings
- DK practised imagery with podiatrists – podiatrists gave feedback on experience.
- DK talked about the impact of the addition of imagery to MI
- Podiatrists practiced role play.
- Summary if session

## Abbreviations

DFD: Diabetes-Related Foot Disease
MI: Motivational Interviewing
FIT: Functional Imagery Training
TIDieR: Template for Intervention Description and Replication
IWGDF: International Working Group on the Diabetic Foot
MITI: Motivational Interviewing Treatment Integrity Tool
FIT-QC: Functional Imagery Training – Quality Coding
